# Carbon Monoxide Improves Cardiac Function and Mitochondrial Population Quality in a Mouse Model of Metabolic Syndrome

**DOI:** 10.1371/journal.pone.0041836

**Published:** 2012-08-01

**Authors:** Steve Lancel, David Montaigne, Xavier Marechal, Camille Marciniak, Sidi Mohamed Hassoun, Brigitte Decoster, Caroline Ballot, Caroline Blazejewski, Delphine Corseaux, Bernadette Lescure, Roberto Motterlini, Remi Neviere

**Affiliations:** 1 EA4484, Physiology Department, Lille 2 University, Lille, France; 2 EA2693, Lille 2 University, Lille, France; 3 INSERM IFR65, Institut de Recherche en Sante Saint Antoine (Irssa), Faculty of Medicine, Paris, France; 4 INSERM U955, Faculty of Medicine, Paris-Est University, Créteil, France; Pennington Biomedical Research Center, United States of America

## Abstract

**Aims:**

Metabolic syndrome induces cardiac dysfunction associated with mitochondria abnormalities. As low levels of carbon monoxide (CO) may improve myocardial and mitochondrial activities, we tested whether a CO-releasing molecule (CORM-3) reverses metabolic syndrome-induced cardiac alteration through changes in mitochondrial biogenesis, dynamics and autophagy.

**Methods and Results:**

Mice were fed with normal diet (ND) or high-fat diet (HFD) for twelve weeks. Then, mice received two intraperitoneal injections of CORM-3 (10 mg.kg^−1^), with the second one given 16 hours after the first. Contractile function in isolated hearts and mitochondrial parameters were evaluated 24 hours after the last injection. Mitochondrial population was explored by electron microscopy. Changes in mitochondrial dynamics, biogenesis and autophagy were assessed by western-blot and RT-qPCR. Left ventricular developed pressure was reduced in HFD hearts. Mitochondria from HFD hearts presented reduced membrane potential and diminished ADP-coupled respiration. CORM-3 restored both cardiac and mitochondrial functions. Size and number of mitochondria increased in the HFD hearts but not in the CORM-3–treated HFD group. CORM-3 modulated HFD-activated mitochondrial fusion and biogenesis signalling. While autophagy was not activated in the HFD group, CORM-3 increased the autophagy marker LC3-II. Finally, *ex vivo* experiments demonstrated that autophagy inhibition by 3-methyladenine abolished the cardioprotective effects of CORM-3.

**Conclusion:**

CORM-3 may modulate pathways controlling mitochondrial quality, thus leading to improvements of mitochondrial efficiency and HFD-induced cardiac dysfunction.

## Introduction

Metabolic syndrome predisposes to cardiovascular diseases. Altered substrate utilization, i.e. reduced glucose utilization and enhanced fatty acid metabolism, and functional and structural alterations of the mitochondrial population are among the important mechanisms involved in this form of myocardial dysfunction [1].

Mitochondrial network dynamics as well as a balance between biogenesis of new mitochondria and autophagy of damaged organelles control quality of the mitochondrial population. Dysregulations of these three pathways may have direct consequences on cardiac function. First, pharmacological inhibition of cardiac mitochondrial fission [2] increased cardiac resistance to ischemia/reperfusion injury. Second, peroxisome proliferator-activated receptor γ coactivator-1α (*Pgc-1α*) cardiac overexpression triggers uncontrolled and excessive biogenesis that may disrupt sarcomeres and may lead to dilated cardiomyopathy [3]. Third, regarding the autophagy process, cardiac specific Atg5-deficient mice display disorganized sarcomeres and mitochondrial accumulation along with myocardial dysfunction [4]. Although cardiac mitochondria biogenesis is stimulated in the context of diabetes and appears to be maladaptive as cardiac energy production remains insufficient [5], these three signalling pathways regulating mitochondria quality are not well characterized in cardiac dysfunction that occurs during metabolic syndrome.

Carbon monoxide (CO) is a well-known poison that binds to haemoglobin with a high affinity. If inhaled at high doses and for prolonged periods of time, CO alters oxygen transport leading to severe tissue hypoxia. In contrast, controlled amounts of CO appear to be beneficial to tissues, as this gas is emerging as an interesting therapeutic agent in a variety of pathophysiological processes [6]. For instance, in the heart, hypoxia triggers CO production by inducing heme oxygenase-1 (HO-1) expression, which in turn may increase cGMP level leading to coronary artery vasodilatation as a cardiac protective response [7]. Studies using transgenic animal models also demonstrated that heme oxygenase overexpression was protective against ischemia/reperfusion injury [8]. Different approaches have been proposed to therapeutically deliver CO. Among them, CO-releasing molecules (CO-RMs) have been developed based on the chemical properties of transition metals [9]. CORM-3, ruthenium-based metal carbonyl, was the first water-soluble compound being characterized [10]. CORM-3 rapidly releases CO in biological fluids without a significant binding to haemoglobin *in vivo* [11]. We previously demonstrated that CORM-3 exerts cardioprotective effects by increasing mitochondrial function and biogenesis during experimental sepsis [12]. Upregulation of HO-1/CO pathway ameliorates insulin sensitivity and glucose tolerance [13]. Therefore, CORMs may improve both cardiac mitochondria function and insulin sensitivity during metabolic syndrome.

In this context, we first characterized the derangement of cardiac and mitochondrial function in a mouse model of metabolic syndrome. Then, we tested whether treatment of mice with CORM-3 would improve metabolic syndrome-induced cardiac dysfunction through changes in mitochondrial activities by primarily assessing dynamics, biogenesis and autophagy. Finally, we tested whether inhibition of autophagy was sufficient to abolish CORM-3-mediated cardioprotective effects in a model of isolated hearts.

## Materials and Methods

### Ethics Statement

All experiments were carried out in accordance with national guidelines and approved by DDSV-NPDC-Lille (Permit Number 59-350206).

### Animal Care and Feeding

Five-week-old C57BL/6 female mice were obtained from Charles River Laboratory (L’Arbresle, France). Mice were randomly divided in two groups: one had free access to a standard chow diet (normal diet ND); the other received a high-fat diet (HFD, D12492, SSNIFF, Soest, Germany) in which 60% of calories were from lard fat. Both groups had free access to water and were kept under a 12 h: 12 h light-dark cycle. Mice were weighed once a week and food intake was monitored daily. After 10–12 weeks of feeding, animals were injected with CORM-3 (10 mg.kg^−1^) as described hereinafter (see “Treatments” paragraph of the current section) and studied 24 hours after the last injection.

### Analysis of Plasma

Blood (100 µl) was withdrawn from tail after an overnight fasting period and glucose concentration was measured with a glucometer (Nova Biomedical, Les Ulis, France). Then, blood was collected into EDTA-coated tubes and centrifuged for 15 min at 1000 *g* at 4°C. The resultant plasma was aliquoted and stored at −80°C. Systemic insulin, adiponectin, leptin levels were determined by the use of Lincoplex immunoassay kits (Millipore, Molsheim, France). True triglycerides were measured with the determination kit from Sigma Aldrich (St Quentin Fallavier, France). Total cholesterol was quantified with the cholesterol RTU and calibrator kits (Biomérieux, Marcy-L’Etoile, France).

### Oral Glucose Tolerance Test (OGTT)

Fasted animals received by gavage a glucose solution (2 mg.g^−1^ total body weight). Glucose levels were measured on blood samples that were taken before glucose administration and 10, 20, 30, 60 and 120 min after glucose ingestion.

### Insulin Tolerance Test (ITT)

Fasted animals received 0.5 mUI.g^−1^ total body weight of insulin diluted in saline solution. Tail blood samples were taken before insulin injection and 15, 30 and 60 minutes later. Glucose concentration was measured using a glucose meter. Mice never presented any troubles or convulsions. In all cases, a glucose solution (200 mg.dl^−1^) was ready for injection if the mice started exhibiting these symptoms.

### Exercise Stress Test

First, mice performed a 10 m.min^−1^ run, for 10 minutes, two days prior the exercise stress test to be acclimatized to the treadmill, as previously described [14]. Then, they were placed onto a single lane treadmill enclosed in a metabolic chamber connected to Oxymax oxygen (O_2_) and carbon dioxide (CO_2_) sensors (Columbus Instruments, Columbus, OH). After 30 min, basal whole body O_2_ uptake (*V*O_2_) and CO_2_ production (*V*CO_2_) were automatically calculated. The basal respiratory exchange ratio (RER), used to determine metabolism of the animal, was the ratio *V*CO_2_ to *V*O_2_. Mice started running on the treadmill at 10 m.min^−1^ on a 0% incline. In order to evaluate maximal oxygen consumption increase, the treadmill speed was incremented every 3 min by 4 m.min^−1^ until mice reached exhaustion, obtained when mice stayed on the electrical shocker plate for 5 sec without trying going back to the treadmill.

### Assessment of Myocardial Function

After cervical dislocation, mouse heart was quickly mounted onto a Langendorff perfusion apparatus. Hearts were perfused at a constant coronary flow (2.5 ml.min^−1^) with non-recirculating Krebs–Henseleit bicarbonate (KHB containing 11 mM glucose) buffer and paced at 9 Hz. Coronary perfusion pressure (CPP) was continuously monitored. When required, 3-methyladenine (10 mmol.l^−1^) was added in the KHB perfusate 15 min before addition of CORM-3 (10 µmol.l^−1^). The acute effects on heart mechanics and oxygen consumption were measured 60 min after CORM-3 addition. Mechanical activity was assessed through isovolumic contraction by inserting into the left ventricle a latex balloon connected to a pressure transducer. The balloon was filled with aqueous solution to achieve a left ventricular end-diastolic pressure of 6–8 mmHg. Pressure transducer was connected to a ML118 bridge amplifier that fed into a Powerlab 8 SP high-performance data acquisition system (ADInstruments Ltd. by Phymep, Paris, France). Contractile performance of the left ventricle was evaluated by developed pressure (LVDP) and its first derivatives.

Oxygen partial pressure was measured in coronary perfusate and effluent using specific electrodes to calculate myocardial O_2_ uptake, MVO_2_ (µL.min^−1^.g^−1^). LVDP - heart rate (HR) product to MVO_2_ ratio was calculated as a surrogate of cardiac efficiency.

### Treatments

*In vivo* treatment: CORM-3, tricarbonylchloroglycinato ruthenium(II) Ru(CO)_3_Cl(NH_2_CH_2_CO_2_), was synthesized as previously described [10]. The compound was solubilized in a saline solution (NaCl 0.9%). Mice received one injection of CORM-3 (10 mg.kg^−1^ total body weight, intraperitoneally, a dose equivalent to approximately 400 µM) at 6 pm and another one at 10 am the day after, as published before [12]. The experiments were then performed at 24, 48 or 72 hours after the second injection. In another set of experiments, mice were injected once a day for five consecutive days with CORM-3 (10 mg.kg^−1^ total body weight) and cardiac function was evaluated the day after. In some experiments, to test the effect of the ruthenium compound, iCORM-3, the inactive form of CORM-3 prepared as previously described [12], was injected twice (10 mg.kg^−1^ total body weight, intraperitoneally) and the effect on heart function was studied 24 hours after the last injection.

*Ex vivo* treatment: in some experiments, isolated hearts from HFD mice were perfused onto a Langendorff apparatus. Then, CORM-3 (10 µmol.l^−1^) was added to the KHB and acute effects on heart mechanics and oxygen consumption were measured 60 min after CORM-3 addition. To test whether autophagy was involved in the effects observed after *ex vivo* treatment with CORM-3, 3-methyladenine (10 mmol.l^−1^) was added in the KHB perfusate 15 min before addition of CORM-3 in the perfusate.

### Determination of Mitochondrial Respiration

Freshly excised heart was placed into ice-cold biopsy preservation solution BIOPS [15]. Fiber bundles were separated and placed into a BIOPS solution containing 50 µg/mL saponin. After 30 min, permeabilized fibers were rinsed three times with mitochondrial respiration media Mitomed 2 [15]. Approximately 4 mg of wet fibers were placed in the oxygraph O2K (Oroboros Instruments, Innsbruck, Austria). First, 10 mmol.l^−1^ glutamate +2 mmol.l^−1^ malate were added into the chambers. The subsequent measured respiration, i.e. in absence of exogenous ADP, is referred to as state 2 [16]. Then, 2.5 mmol.l^−1^ ADP were brought to obtain the ADP-coupled respiration called state 3. As in permeabilized fibers state 4 respiration cannot be achieved because of the presence of many intracellular ATPases that avoid total ADP depletion, the ratio state 3 to state 2 was used to evaluate the quality of the mitochondrial coupling between oxygen consumption and phosphorylation [17].

### Measurements of Mitochondrial Membrane Potential and Calcium Retention Capacity

Cardiac mitochondria were isolated as previously described [18]. 400 µg of mitochondria were placed into a multi-port chamber equipped with tetraphenylphosphonium (TPP^+^) and calcium -selective microelectrodes and reference electrodes (WPI, Sarasota, FL, USA). Mitochondria were gently stirred for 90 sec in the assay buffer [18] containing 1.5 µmol.l^−1^ TPP^+^. Calcium pulses (10 µmol.l^−1^) were added every 90 sec until a massive calcium release was observed. Calcium retention capacity was defined as the total amount of calcium added until permeability transition. Transmembrane potential ΔΨm was calculated as 59log(v/V) - 59log(10Δ^E/59^−1), where v is matrix volume (1.1 µl.mg^−1^ mitochondrial protein), V is volume chamber (1 ml), and ΔE is voltage difference before and after calcium-induced permeability transition, expressed in millivolts. Purity and integrity of isolated mitochondria were assessed by measuring specific activities of nicotinamide adenine dinucleotide phosphate NADPH-cytochrome c reductase, as an endoplasmic reticulum marker enzyme, and cytochrome c oxidase, as an inner mitochondrial membrane marker enzyme.

### Quantitative RT-PCR

Total RNAs from flash frozen heart tissue were extracted using TriZol reagent (Life Technologies SAS, Villebon sur Yvette, France) and purified with the PureLink RNA mini kit (Life Technologies SAS) according to manufacturer’s instructions. Total RNAs (1 µg) were reverse-transcribed using transcriptor first strand cDNA synthesis kit (Roche Applied Science, Meylan, France). Real-time RT-PCR was performed using an Eppendorf Realplex S2 (Eppendorf, Le Pecq, France) and Mesa Blue qPCR Master Mix Plus for SYBR green assay (Eurogentec, Angers, France). Primers used for the real-time RT-PCR are summarized in Table S1. β-actin was used as reference. Realplex software was used to quantify differences in gene expression. Results are expressed in fold expression compared to the ND group.

### Western-blotting

Flash frozen hearts were homogenized at 4°C in RIPA buffer (in mmol.l^−1^: Tris 10, NaCl 140, EDTA 5, PMSF 1, with Triton X-100 1%, Deoxycholate 1%, SDS 0.1%, and in µg.ml^−1^: aprotinin 10, leupeptin 10, pepstatin 10, at pH 7.4) using a glass tissue grinder. After a 15 min-centrifugation at 15,000 *g* at 4°C, supernatants were collected and stored at −80°C until use. Proteins (50 µg) were resolved by SDS-PAGE, transferred onto a nitrocellulose membrane and incubated with the following antibodies: anti-UCP3 rabbit polyclonal antibody (1/1000; Thermo Fisher Scientific, Brebieres, France), anti-GAPDH rabbit monoclonal antibody (1/5000; Cell Signaling Technology, Beverly, MA, USA), anti-PGC1-α rabbit polyclonal antibody (1/1000; Cell Signaling Technology), anti-LC3 rabbit polyclonal antibody (1/500; Abcam, Paris, France). Blots were developed with ECL Plus reagent (GE Healthcare, Templemars, France).

### Electron Microscopy

Cardiac free wall was fixed in a formaldehyde-glutaraldehyde-picric acid solution. Once embedded, ultrathin sections were observed under a Zeiss EM 902 electron microscope, at a 7000X magnification. Mitochondrial morphometric analysis was performed on four different sections per heart. Each picture was analyzed by superimposing a grid of 144 µm^2^ made of lines spaced by 1 µm. The number of grid intersections superimposing mitochondria was scored and interpreted as mitochondrial volume density. In addition, surface of each individual mitochondrion was measured by the use of Gatan Digital Micrograph software (Gatan Inc, Pleasanton, CA, USA).

### Statistical Analysis

Data are presented as means ± S.E.M. Statistical analysis was carried out using two-tailed unpaired t-test when comparing two groups and ANOVA when comparing more than two groups. Analysis was performed on GraphPad Prism software 5.0 (San Diego, CA, USA). Differences were considered significant when p<0.05.

## Results

### High-fat Diet Led to Metabolic Syndrome and Cardiac Dysfunction

At the onset of the regimen, mouse body weight was identical in both normal diet (ND) and high-fat diet (HFD) groups (Table S2). Over time, the body weight increased in both groups but after 2 months of feeding, the gain of weight was about 53% in the HFD compared to 15% in the ND mice (Table S2). Fasting glucose, insulin, cholesterol, triglycerides and leptin circulating levels increased significantly in the HFD mice compared to ND animals while blood adiponectin concentration was reduced (Table S2). Taken together, these data indicate that HFD mice develop a phenotype resembling to the human metabolic syndrome.

**Figure 1:**
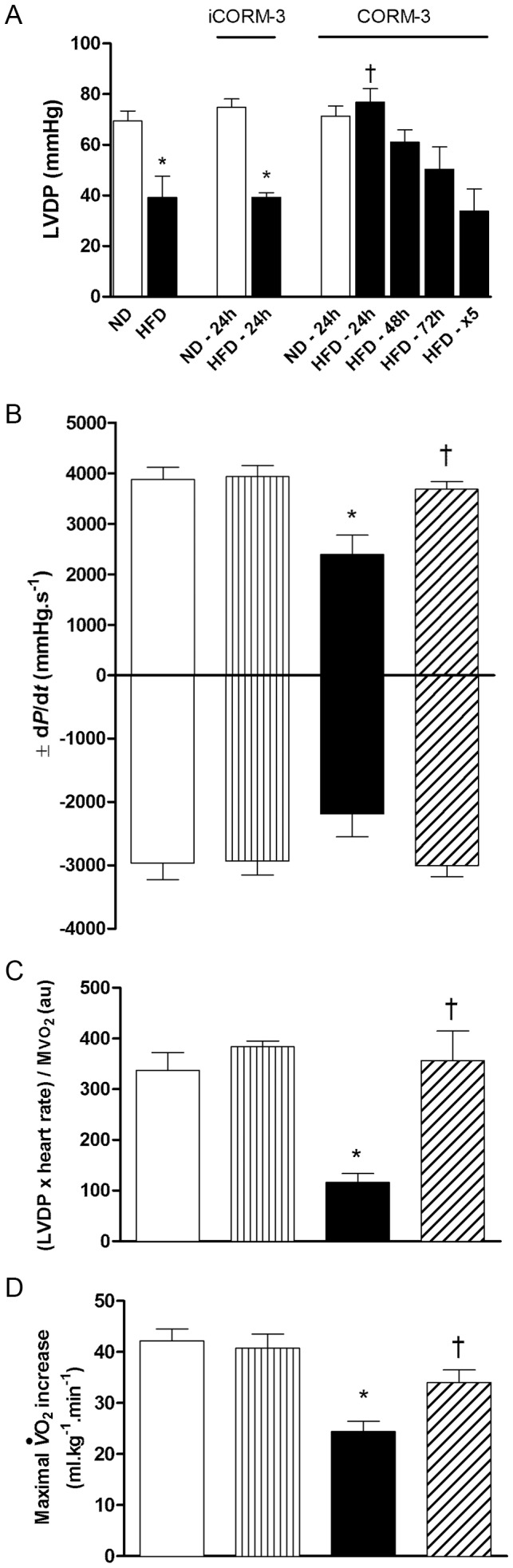
Effects of CORM-3 on high-fat-induced cardiac dysfunction. (A) Left Ventricular Developed Pressure (LVDP) of hearts isolated from normal diet (ND) or high-fat diet (HFD) mice injected twice with iCORM-3 (10 mg.kg^−1^, intraperitoneally, studied 24 hours after the second injection) or CORM-3 (10 mg.kg^−1^, intraperitoneally, studied 24, 48 or 72 hours after the second injection, or 24 hours after the fifth injection (x5); see Materials and Methods). (B) LVDP first derivatives ±d*P*/d*t* and (C) ratio (LVDP x Heart Rate)/MVO_2_ (see Materials and Methods) obtained from ND, ND + CORM-3, HFD and CORM-3–treated HFD mouse hearts. Evaluation was performed 24 hours after the second injection. (D) Maximal oxygen uptake (*V*O_2_) increase of mice subjected to exercise stress test. Data represent means ± SEM. n  = 5–8 in each group. White bars  =  ND; vertically hatched bars  =  ND + CORM-3; black bars  =  HFD; crosshatched bars  =  HFD + CORM-3. * p<0.05 vs. ND, † p<0.05 vs. HFD.

Intrinsic contractile function was evaluated on isolated hearts. Left ventricular developed pressure (LVDP) was significantly decreased in HFD mice as compared with ND animals (Table S2, Figure 1A). There were no differences in the coronary perfusion pressure (CPP) between groups (Table S2).

### CORM-3 Ameliorated Cardiac Function in Mice with Metabolic Syndrome

Compared to HFD, injection of CORM-3 (10 mg.kg^−1^) for 5 consecutive days did not improve cardiac function (Figure 1A). Next, we injected mice twice with CORM-3 (see Materials and Methods) and heart function was evaluated 24, 48 or 72 hours after the last injection. Compared to untreated HFD mice, LVDP was greatly improved in hearts isolated from HFD mice that were studied 24 hours after the last injection of CORM-3 (Figure 1A). Beneficial effect of CORM-3 on cardiac function lessened overtime (Figure 1A). iCORM-3 injections (see Materials and Methods) did not alter LVDP neither in ND mice, nor in HFD mice (Figure 1A).

Consequently, we performed all subsequent experiments in CORM-3 -treated animals 24 hours after the last injection (HFD + CORM-3). In addition to LVDP, CORM-3 also ameliorated d*P*/d*t* values compared to HFD hearts (Figure 1B). Ratio (LVDP × HR) to MVO_2_, a proxy measure for cardiac efficiency (see Materials and Methods), was reduced in the HFD hearts and rescued by CORM-3 (Figure 1C). CORM-3 injections in ND mice had no effects (Figure 1).

Cardiac output reduction may limit the rate of oxygen exchange to the tissues and would in turn restrict performance to exercise. Thus, we subjected mice to a maximal physical exercise and measured their maximal oxygen consumption (*V*O_2_) increase as a surrogate of the cardiovascular adaptation *in vivo*. The increase in *V*O_2_ consecutive to exercise was reduced in HFD mice as compared with ND littermates (Figure 1D). However, *V*O_2_ increase was significantly improved in HFD + CORM-3 group compared to HFD mice (Figure 1D).

### CORM-3 did not Reverse Metabolic Syndrome but Improved Maximal Aerobic Capacity

**Figure 2:**
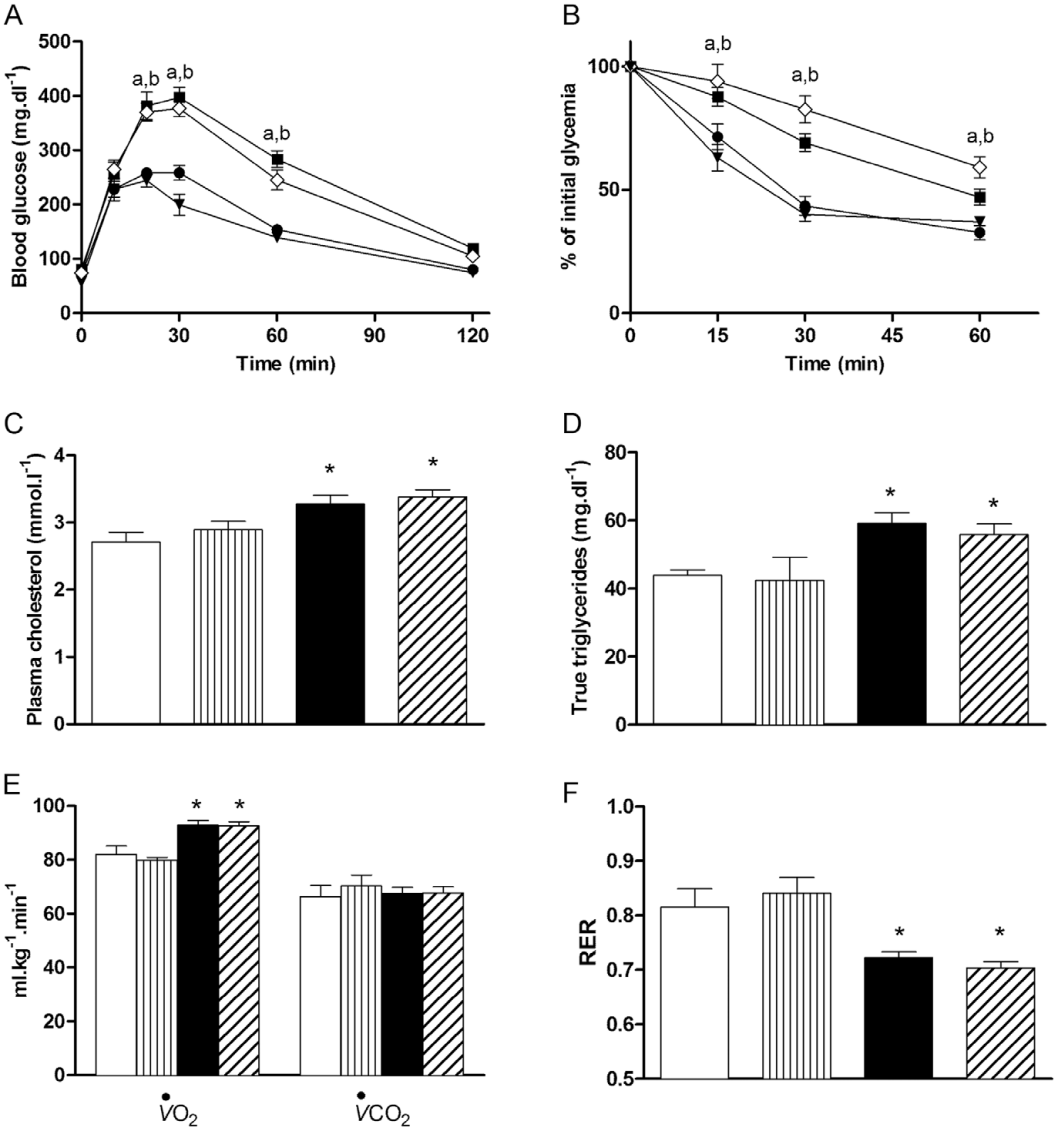
Effects of CORM-3 on metabolic parameters. (A) Oral glucose tolerance and (B) insulin tolerance tests were performed on ND (black circles), ND + CORM-3 (black triangles), HFD (black squares) and HFD + CORM-3 (white diamonds) mice. Data are means ± SEM. n  = 10 in each group. (a) p<0.05 between HFD and ND; (b) p<0.05 between HFD + CORM-3 and ND. (C) Plasma cholesterol and (D) true triglyceride measurements were performed on 6–10 different samples in each group. Data are means ± SEM. (E) Basal oxygen uptake (*V*O_2_) and carbon dioxide rejection (*V*CO_2_), (F) respiratory exchange ratio (RER). Data represent means ± SEM. n  = 5–7 in each group, * p<0.05 vs. ND. White bars  =  ND; vertically hatched bars  =  ND + CORM-3; black bars  =  HFD; crosshatched bars  =  HFD + CORM-3.

As high-fat diet led to metabolic syndrome and cardiovascular dysfunction, we tested whether the beneficial effects mediated by CORM-3 were related to changes in metabolic parameters. Neither glucose tolerance (Figure 2A) nor insulin resistance (Figure 2B) was restored by CORM-3. Similarly, CORM-3 was unable to reverse plasma cholesterol (Figure 2C) and true triglyceride (Figure 2D) elevations triggered by the high-fat regimen.

Then, we measured oxygen uptake (*V*O_2_) and carbon dioxide rejection (*V*CO_2_) in mice under basal condition. While no differences in *V*CO_2_ were observed between the three groups (Figure 2E), mice fed with HFD consumed more oxygen than ND mice (Figure 2E). CORM-3 injection did not modify oxygen uptake (Figure 2E). As a consequence, HFD and HFD + CORM-3 mice used preferentially lipids as their respiratory exchange ratios (RER) were close to 0.7 (Figure 2F). On the contrary, RER value of ND mice was about 0.8, indicating that relative substrate utilization between lipids and carbohydrates was different from HFD and HFD + CORM-3 mice. Injection of CORM-3 in ND mice had no effects on all the above parameters (Figure 2).

### CORM-3 Ameliorated Cardiac Mitochondrial Function of HFD Mice

**Figure 3:**
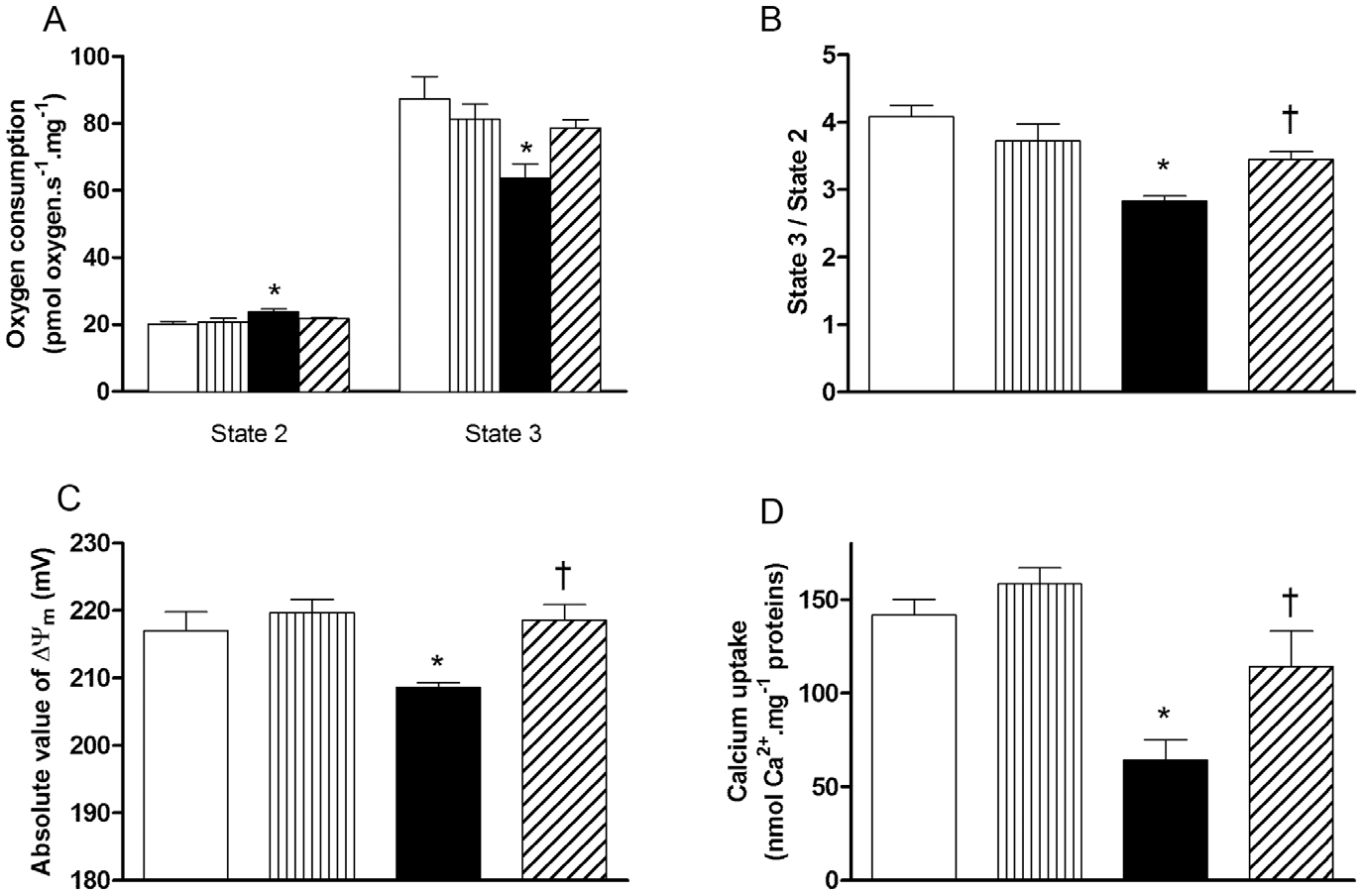
CORM-3 improved mitochondrial function. (A) Oxygen consumption of permeabilized fibers in presence of glutamate and malate without exogenous ADP (state 2) or with 2.5 mM ADP (state 3) and (B) state 3 to state 2 ratio. Mitochondrial membrane potential (C) and calcium retention capacity (D) measured on isolated cardiac mitochondria. White bars  =  ND; vertically hatched bars  =  ND + CORM-3; black bars  =  HFD; crosshatched bars  =  CORM-3–treated HFD mice. Data are means ± SEM. n  = 6–8 in each group. * p<0.05 vs. ND, † p<0.05 vs. HFD.

Because of the positive effects of CORM-3 on (LVDP x HR) to MVO_2_ ratio (Figure 1C) and maximal *V*O_2_ increase (Figure 1D), we evaluated function of cardiac mitochondria. First, mitochondrial respiration was evaluated on saponin-skinned cardiac fibers. Oxygen consumption stimulated by glutamate and malate in absence of exogenous ADP, so-called state 2 [16], was higher in HFD compared to ND mice (Figure 3A). On the contrary, ADP-coupled respiration, corresponding to state 3, was reduced in HFD compared to ND mice (Figure 3A). As a result, state 3 to state 2 ratio of HFD animals was reduced by 30% compared to ND littermates (Figure 3B). No differences were detected between ND and HFD + CORM-3 for both states 3 and 2 (Figure 3A), hence a better state 3/state 2 ratio for HFD + CORM-3 vs. HFD (Figure 3B).

**Figure 4:**
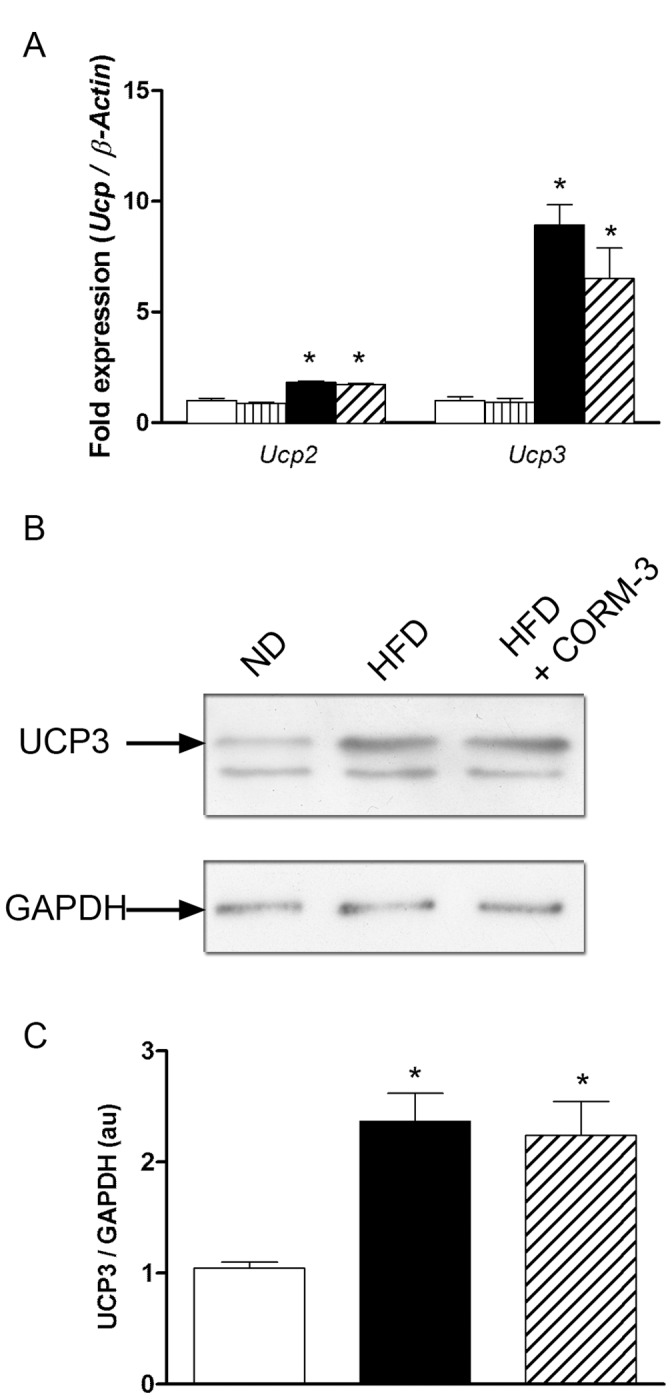
CORM-3 did not change UCP expression. (A) Results of quantitative RT-PCR detecting *Ucp2* and *Ucp3* mRNA expressions. Results were normalized to *â-actin*. (B) Representative western-blot targeting UCP3 and GAPDH proteins and (C) densitometric analysis of UCP3 to GAPDH ratio. White bars  =  ND; vertically hatched bars  =  ND + CORM-3; black bars  =  HFD; crosshatched bars  =  CORM-3–treated HFD mice. Data are means ± SEM. n  = 5 in each group. * p<0.05 vs. ND.

To test potential intrinsic mitochondrial defects, we isolated mitochondria to measure transmembrane potential ΔΨm as well as calcium retention capacity. Compared to ND, mitochondria from HFD hearts displayed a reduced ΔΨm (Figure 3C) as well as a two-fold decrease in calcium retention capacity (Figure 3D). CORM-3 ameliorated both parameters (Figure 3, C-D). CORM-3 -induced mitochondrial function improvement was not due to changes in uncoupling protein expression (Figure 4). Indeed, compared to ND mice, both *Ucp2* and *Ucp3* mRNA expressions were increased in HFD and remained elevated in the HFD + CORM-3 group (Figure 4A). In addition, UCP3 protein expression doubled in HFD and HFD + CORM-3 compared to ND (Figure 4, B-C). None of these mitochondrial parameters were affected by CORM-3 injection in ND mice (Figure 4). Because of the lack of effect of CORM-3 in ND mice on myocardial and mitochondrial functions, we focused our study on pathways regulating mitochondrial population quality in the ND, HFD and HFD + CORM-3 groups.

### CORM-3 Modulated Pathways Controlling Mitochondrial Quality

**Figure 5:**
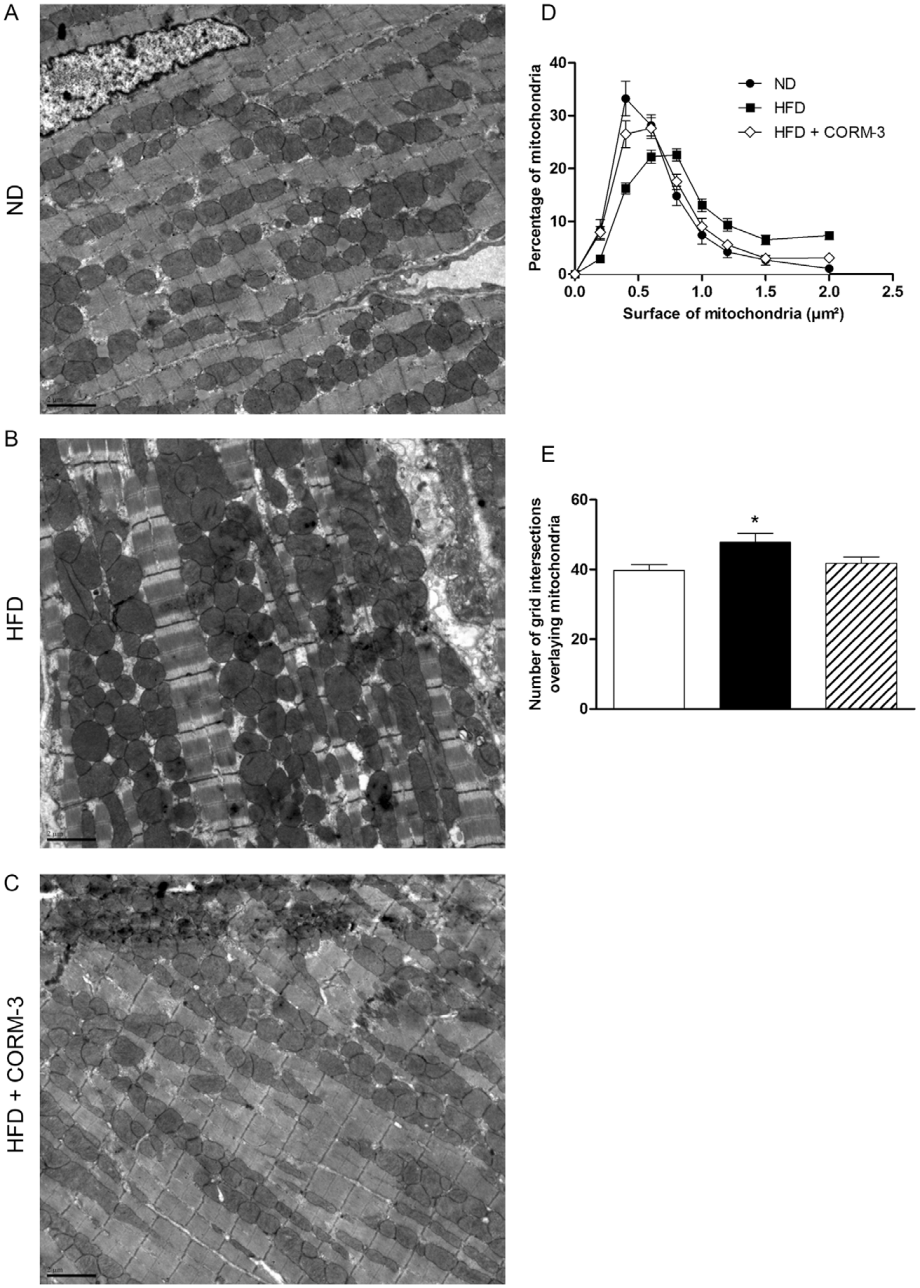
CORM-3 modified cardiac mitochondrial population. (A) Representative electron micrographs of ND, HFD and HFD + CORM-3 mouse hearts. Magnification x7000, scale bar  = 2 µm. (B) Surface of every single mitochondrion from three randomized tissue sections prepared from four hearts in each group was measured. Black circles: ND; black squares: HFD; white diamonds: HFD + CORM-3. Results are expressed in percentage. (C) A grid of 144 µm^2^ was positioned onto each micrograph (3 sections per heart, 4 hearts per group) and the number of intersections with mitochondria was scored. White bars  =  ND; black bars  =  HFD; crosshatched bars  =  CORM-3–treated HFD mice. Data are means ± SEM. * p<0.05 vs. ND.

Electron microscopy on cardiac sections revealed that HFD hearts had bigger mitochondria compared to ND hearts (Figure 5, A-B). Number of grid intersections superimposing mitochondria was higher in the HFD compared to ND group (Figure 5, A and C), indicating that mitochondria were either more numerous and/or bigger. In addition, morphometric analysis revealed that HFD mice treated with CORM-3 displayed a mitochondrial population similar to ND hearts (Figure 5, A-C).

**Figure 6:**
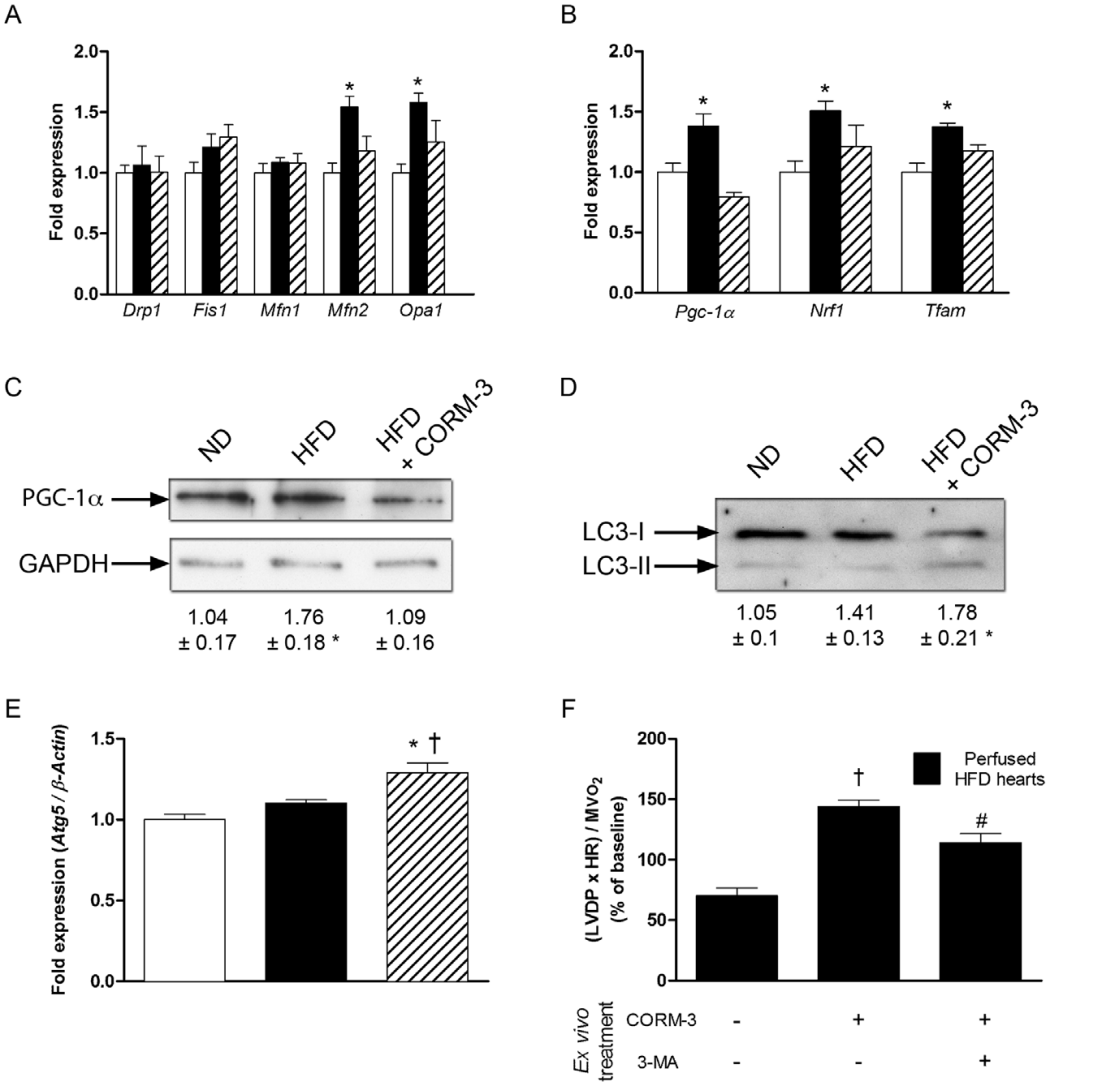
Effects of CORM-3 on pathways controlling mitochondrial quality. RT-qPCR results of fusion/fission (A) and biogenesis (B) -related transcripts in cardiac tissue obtained from ND (white bars), HFD (black bars) or HFD + CORM-3 (crosshatched bars) mice. Data were normalized to *â-actin*. (C) Representative western-blot of PGC-1α and GAPDH in ND, HFD and HFD + CORM-3 mouse hearts. Values are results of densitometric analysis normalized to GAPDH. (D) Representative western-blot of LC3 proteins in ND, HFD and HFD + CORM-3 mouse hearts. LC3-I represents the unactivated form; LC-II is the activated protein that has been lipidated. Numbers represent the pro-autophagy ratio LC3-II to LC3-I obtained after densitometric analysis. (E) *Atg5* RT-qPCR results normalized to *â-actin*. White bars  =  ND; black bars  =  HFD; crosshatched bars  =  CORM-3–treated HFD mice. Data are means ± SEM. n  = 5 in each group. * p<0.05 vs. ND, † p<0.05 vs. HFD. (F) Ratio (LVDP x Heart Rate)/MVO_2_ of isolated perfused heart from HFD mice. HFD hearts were pretreated or not with 10 mM 3-methyladenine (3-MA) for 15 min and then perfused with Krebs-Henseleit buffer containing 10 µM of CORM-3. Measurement was performed 60 min after CORM-3 perfusion. Black bars: isolated and perfused HFD hearts. Data are means ± SEM. n  = 4–5 in each group. † p<0.05 vs. untreated HFD hearts, # p<0.05 vs. CORM-3 treated HFD hearts.

We then explored mitochondrial dynamics, biogenesis and autophagy. Fission-related transcripts, *Drp1* and *Fis1*, did not change in any groups (Figure 6A). While *Mfn1* remained unchanged, fusion-related mRNAs, *Mfn2* and *Opa1*, increased by 50% in mice fed with high-fat regimen (Figure 6A). CORM-3 partially reduced high-fat-induced elevation in *Mfn2* and *Opa1* mRNA expression (Figure 6A).

*Pgc-1α* mRNA and protein expressions were higher in the HFD group compared to ND (Figure 6, B-C). Downstream transcription factors *Nrf1* and *Tfam* were upregulated in the HFD compared to ND (Figure 6B). CORM-3 abrogated HFD-induced increases in *Pgc-1α*, *Nrf1* and *Tfam* expression (Figure 6, B-C).

Autophagy also participates in the quality control of mitochondrial population. While no differences were observed between ND and HFD, LC3-I protein lipidation into the active form LC3-II was increased in the HFD + CORM-3 group (Figure 6D). Consistently, expression of *Atg5* was significantly increased after CORM-3 injection in HFD animals compared to ND and HFD mice (Figure 6E).

CORM-3 injection, *in vivo*, in HFD mice was associated with both autophagy activation and improved cardiac function. We used 3-methyladenine, an autophagy inhibitor, on isolated HFD heart treated *ex vivo* by CORM-3 (see Materials and Methods) to test whether blocking defective mitochondria removal impeded improvement of cardiac function triggered by carbon monoxide. Addition of CORM-3 in the perfusate of HFD hearts led to an improvement of (LVDP × HR) to MVO_2_ ratio (Figure 6F). Importantly, pretreatment with 3-methyladenine abolished acute CORM-3 effects on myocardial function (Figure 6F).

## Discussion

In the present study, we report that mice fed with high-fat diet developed glucose intolerance, insulin resistance, dyslipidemia, changes in adipocytokines levels and cardiac dysfunction. We further observed that myocardial contractile alterations were related to a poorer quality of the mitochondrial population. More importantly, we report for the first time that delivery of carbon monoxide by CORM-3 significantly attenuated HFD-induced cardiac dysfunction through improvement of mitochondrial function that may also involve elimination of defective mitochondria by autophagy activation.

Consistently with published works [19], we found that three months after the onset of high-fat regimen, mice developed major abnormalities resembling to human metabolic syndrome such as glucose intolerance, insulin resistance, elevated glucose, insulin and leptin circulating levels and reduced adiponectin blood concentration. We also found that HFD led to intrinsic ventricular contractile dysfunction. Interestingly, carbon monoxide delivered by CORM-3 improved cardiac function but only transiently as improved function was only observed at 24 hours after the last injection. A possible explanation could be related to the half-life of carbon monoxide in the organism. Indeed, half-time of CO elimination is about four hours while breathing room-air [20]. On the other side, five consecutive injections may trigger a reduction in cardiac function. This is consistent with previous studies reporting that chronic CO exposure may induce cardiac hypertrophy [21] and arrhythmia [22]. Plus, five consecutive injections may result in elevated blood concentration of CO, mimicking the well-known poisonous effect on oxygen delivery to tissues or inhibition of mitochondrial cytochrome oxidase, leading to a reduced production of ATP [9]. As a consequence, although CORM-3 has striking beneficial effects when injected twice, it remains a molecule that has to be handle with care and that needs further investigation before its uses as a therapeutic agent.

In association with improvement of cardiac function, the ratio (LVDP x HR) to MVO_2_, a surrogate of cardiac efficiency, and the increase in maximal oxygen consumption were higher in the CORM-3-injected group compared to untreated HFD mice. First, these results are in agreement with those obtained in genetically modified mice [5]. Second, as improvement of both cardiac function and animal oxygen uptake were not related to changes in metabolism, carbon monoxide may have changed the cellular redox state [23], reduced the pro-inflammatory state [24] or exerted cytoprotective effects on the myocardium [25]. Here, we rather explored mitochondrial function as it may also contribute to the improvement of myocardial function.

We used multiple approaches to characterize mitochondrial function. First, respiration measured from cardiac skinned fibers was lower when isolated from HFD animals. Second, mitochondrial membrane potential and calcium retention capacity of isolated mitochondria were reduced in the HFD group. In all cases, CORM-3 exerted beneficial effects. Although carbon monoxide has been reported to exert beneficial effects on mitochondria through production of cGMP and reactive oxygen species and activation of Akt [26], a clear understanding of the underlying molecular mechanisms is still lacking and warrants additional studies. Here, we further assessed whether carbon monoxide improved mitochondrial function through changes in mitochondrial population (density and size of mitochondria) and intrinsic mitochondrial properties (oxidative capacity and uncoupling level). Boudina et al. [5] suggested that cardiac UCP increase in diabetic hearts might participate to mitochondrial intrinsic dysfunction. In our model, we also detected increases in *Ucp2* and *Ucp3* but CORM-3 did not change their expression. Although we cannot rule out the fact that CORM-3, in a time-dependent manner, may directly modulate uncoupling and UCP activity in mitochondria [23], the ultimate effects of CORM-3 originating from the present study can be explained by an overall improvement of mitochondrial population quality.

Electron microscopy revealed that HFD led to an increase in both mitochondrial size and number, two parameters modulated by CORM-3. Moreover, *Mfn2* and *Opa1* expressions increased in the HFD group. To our knowledge, this is the first time that such an observation has been made in cardiac tissue in the context of metabolic syndrome, diabetes or obesity. Physiological significance of such modifications in maintaining myocardial energy homeostasis remains largely unknown in the heart and requires further studies. In skeletal muscle, repression of *Mfn2* reduces glucose oxidation, mitochondrial respiration and potential. Rhodes *et al.* [27] report that CO inhalation leads to increase in pro-fusion protein expression in biopsies of the *vastus lateralis* muscle.

PGC-1α expression at both mRNA and protein levels was elevated in HFD mice. This observation is consistent with studies that reported mitochondrial proliferation in the *db*/*db* mice [5] and also in cardiac biopsies from diabetic patients [28]. CORM-3 blocked the HFD-induced elevations in *Pgc-1α*, *Nrf1* and *Tfam* while other reports have found that CO activated mitochondrial biogenesis [29]. One hypothesis that could reconcile these apparent contradictory findings is that, because of a better mitochondrial function consecutive to CORM-3 treatment, mitochondrial biogenesis would not be required any longer.

A balance between biogenesis and mitochondria autophagy, process known as “mitophagy”, tightly controls the quality of mitochondrial population. An autophagy hallmark is the conjugation of microtubule-associated light chain-3 (LC3-I) to phosphatidylethanolamine (LC3-II), consecutively to the action of a complex constituted of several proteins including Atg5 [30]. Here, while there were no differences between ND and HFD groups, *Atg5* expression and LC3-II to LC3-I ratio increased after treatment with CORM-3. Although additional studies are needed to understand the underlying mechanisms, consistent data from Lee *et al.* described that exposure of human alveolar adenocarcinoma epithelial cells and human bronchial epithelial cells to CO gas also triggered autophagy activation [31]. As a proof of concept, we performed *ex vivo* experiments in which 3-methyladenine, a pharmacological inhibitor of autophagy was administered. We found that 3-methyladenine prevented the positive effects of CORM-3 on cardiac efficiency within one hour, suggesting that CO may improve mitochondrial function through rapid and critical activation of autophagy. Although this striking effect may be questionable as changes in cardiac efficiency occurred within one hour of treatment, a 68% reduction in mitochondrial number consecutive to a 3.5-hour starvation has been observed in HL1 cardiac cell line [32].

In conclusion, improvement of mitochondrial population quality by CORM-3 is associated with a better mitochondrial efficiency, leading to amelioration of HFD-induced cardiac dysfunction. Although these results bring new therapeutic insight, further pharmacokinetic and toxicity studies on CORM-3 are required before administration in humans.

## Supporting Information

Table S1 Primers for RT-qPCR experiments. F: forward, R: reverse.
(DOC)

Table S2 Model features obtained after ten weeks on specific diets. Plasmatic parameters were measured after a 12 hr-fasting period. n = 9–10 in each group. Left ventricular developed pressure (LVDP) and coronary perfusion pressure (CPP) were evaluated on five animals in each group. *P<0.05 vs. normal diet.
(DOC)
