# Supplementary material for: Carbon Monoxide Improves Cardiac Function and Mitochondrial Population Quality in a Mouse Model of Metabolic Syndrome
Source: PLoS One. 2012 Aug 1;7(8):e41836. doi: 10.1371/journal.pone.0041836 (PMC3411569; doi:10.1371/journal.pone.0041836)
Supplement: Table S1 — Primers for RT-qPCR experiments. F: forward, R: reverse. (DOC) [file pone.0041836.s001.doc]

| Accession number | Gene / Gene name | Sequence of primers (5’-3’) |
| --- | --- | --- |
| NM_011671.4 | *Ucp2 / uncoupling protein 2* | F : GTAGCCCAGCCTACAGATGTGG  R : CCTTCCTCTCGTCGAATGGTC |
| NM_009464.3 | *Ucp3 / uncoupling protein 3* | F : GTCTGCCTCATCAGGGTGTT  R : CCTGGTCCTTACCATGCAGT |
| NM_001164226.1 | *Nrf1 / nuclear respiratory factor 1* | F : CCACATTACAGGGCGGTGAA  R : AGTGGCTCCCTGTTGCATCT |
| NM_008904.2 | *Pgc1-α / peroxisome proliferator-activated receptor, gamma, coactivator 1 alpha* | F : CGGAAATCATATCCAACCAG  R : TGAGGACCGCTAGCAAGTTTG |
| NM_009360.4 | *Tfam / mitochondrial transcription factor A* | F : AGCCAGGTCCAGCTCACTAA  R : AAACCCAAGAAAGCATGTGG |
| NM_007393.3 | *β-Actin / β-actin* | F : AGCTGCCTGACGGCCAGGTC  R : GCTCAGGAGGAGCAATGATC |
| NM_152816.2 | *Drp1 / dynamin-related protein 1* | F : CGGTTCCCTAAACTTCACGA  R : GCACCATTTCATTTGTCACG |
| NM_025562.3 | *Fis1 / mitochondrial fission protein 1* | F : CCGGCTCAAGGAATATGAAA  R : CCATGCCTACCAGTCCATCT |
| NM_024200.4 | *Mfn1 / mitofusin 1* | F : GCACAGAGGGTGCTGCTCGG  R : TGGGCTGCATTATCCGGGGC |
| NM_133201 | *Mfn2 / mitofusin 2* | F : GGGGCCTACATCCAAGAGAG  R : GCAGAACTTTGTCCCAGAGC |
| NM_133752.2 | *Opa1 / optic atrophy 1* | F : GATGACACGCTCTCCAGTGAAG  R : CTCGGGGCTAACAGTACAACC |
| NM_053069.5 | *Atg5 / autophagy-related gene 5* | F : GATGGACAGCTGCACACACT  R : TTGGCTCTATCCCGTGAATC |

Table S1
