# Supplementary material for: Carbon Monoxide Improves Cardiac Function and Mitochondrial Population Quality in a Mouse Model of Metabolic Syndrome
Source: PLoS One. 2012 Aug 1;7(8):e41836. doi: 10.1371/journal.pone.0041836 (PMC3411569; doi:10.1371/journal.pone.0041836)
Supplement: Table S2 — Model features obtained after ten weeks on specific diets. Plasmatic parameters were measured after a 12 hr-fasting period. n = 9–10 in each group. Left ventricular developed pressure (LVDP) and coronary perfusion pressure (CPP) were evaluated on five animals in each group. *P<0.05 vs. normal diet. (DOC) [file pone.0041836.s002.doc]

|  | Normal Diet | High Fat Diet |
| --- | --- | --- |
| Initial body weight (g) | 15.9 ± 0.3 | 15.4 ± 0.6 |
| Final body weight (g) | 18.4 ± 0.3 | 24.7 ± 0.5* |
| Glucose (mg.dl^-1^) | 74 ± 3 | 96 ± 7* |
| Insulin (pg.ml^-1^) | 387 ± 74 | 801 ± 105* |
| Leptin (ng.ml^-1^) | 0.6 ± 0.2 | 7.3 ± 1.5* |
| Adiponectin (µg.ml^-1^) | 15.7 ± 1.1 | 13.3 ± 0.5* |
| True triglycerides (mg.dl^-1^) | 44 ± 2 | 59 ± 3* |
| Cholesterol (mmol.l^-1^) | 2.7 ± 0.2 | 3.3 ± 0.1* |
| LVDP (mmHg) | 69.3 ± 4.0 | 43.1 ± 8.4* |
| CPP (mmHg) | 103 ± 28 | 92 ± 23 |

Table S2
